# Editorial: Macromolecular Structure Underlying Recognition in Innate Immunity

**DOI:** 10.3389/fimmu.2018.00980

**Published:** 2018-05-15

**Authors:** Uday Kishore

**Affiliations:** Biosciences, College of Health and Life Sciences, Brunel University London, Uxbridge, United Kingdom

**Keywords:** pattern recognition, innate immunity, host–pathogen interactions, protein–protein interaction, HIV-1, malaria, zebrafish model system

**Editorial on the Research Topic**

**Macromolecular Structure Underlying Recognition in Innate Immunity**

Our innate immune system has evolved to distinguish between self, non-self, altered self, and intrinsic as well as extrinsic danger signals. Recognition is mediated *via* interactions between pattern recognition receptor molecules (PPRs) and their ligands, which include hydrophobic and electrostatic interactions between amino acid residues on the PPRs and uncharged or charged groups on amino acid residues, sugar rings, or DNA/RNA molecules. These PRRs can be phagocytic, sensors, and humoral. Recognition in innate immunity can involve interaction between many ligands with one receptor molecule, and the density and the number of glycans, charge patterns or epitopes dictate a strong and specific recognition, distinct from weak non-specific binding (1). In the case of toll-like receptors (TLRs) and nucleotide-binding oligomerization domain-like receptors (NLRs), the ligand recognition is followed by oligomerization of the receptor molecules. This special topic issue has made an effort to somewhat highlight the complexity of such biological interactions and their, sometimes unexpected, consequences.

Bessa Pereira et al. have taken up the case of a scavenger receptor, SSc5D, which belongs to the scavenger receptor cysteine-rich (SRCR) family, and have made an effort to localize domains involved in pathogen-associated molecular patterns (PAMPs) recognition. SRCR proteins can be membrane-bound or secreted that contain type I macrophage scavenger receptor domain/s. SSc5D is a soluble SRCR composed of an N-terminal, five SRCR domains (N-SSc5D) and a C-terminal mucin-like domain. Based on surface plasmon resonance-measurements of interaction kinetics, N-SSc5D was found to have a better ability to bind *Escherichia coli* strains RS218 and IHE3034, and *Listeria monocytogenes*. This paper speculates on the importance of differential SRCR binding on the strain specificity. An associated commentary on this paper by Lozano and Martínez-Florensa, however, sounds a cautionary note on the interpretation of the data, especially with respect to CD6 that does not seem to be functional in the studies by Bessa Pereira et al. The scientific exchange between the two research groups (Lozano and Martínez-Florensa; Oliveira and Carmo) raises an important issue that a number of variations reported in the literature could owe to the design of the constructs and choice of domains used.

The study and critique on SRCR (Bessa Pereira et al.; Lozano and Martínez-Florensa; Oliveira and Carmo) are followed by some excellent, though diverse, papers that highlight the importance of understanding protein–ligand and protein–protein interactions in innate and adaptive immunity. Two papers (Hellmuth et al.; Wan et al.) dwell upon the modalities of modulating intracellular PRRs (sensors) and their immunological consequences. Hellmuth et al. have made a synthetic bid to impede TLR7 activation by conjugating single-stranded RNA (ssRNA for TLR7 stimulation), small molecules TLR7 agonists (smTLRa for immune potentiation of candidate vaccines), and an interference RNA (siRNA). Contrary to an expected synergistic effect, the authors reported a reduced TLR7 activation and encouraged the notion of shielding effect of the conjugates on TLR7 stimulation (Hellmuth et al.). Wan et al. have examined, using a grass carp model, downstream signaling and interferon (IFN) response involving two intracellular sensors: retinoic acid-inducible gene I (RIG-I) and melanoma differentiation-associated gene 5 (MDA5). Following grass carp reovirus infection, MDA5 induced an elaborate type I IFN response; both MDA5 and RIG-I facilitated the total phosphorylation levels of IFN regulatory factor (IRF) 3 and 7 (IRF7). Whereas MDA5 enhanced the heterodimerization of IRF3 and IRF7 and homodimerization of IRF7, RIG-I facilitated the heterodimerization and attenuated IRF7 homodimerization. The consequence of this differential multimerization renders MDA5 a more effective modulator of IFN response compared to RIG-1 (Wan et al.). In an exciting paper on zebrafish innate immunity, García-Valtanen et al. have investigated, *via* transcriptomics and immunohistochemistry, the immune responses of *Rag1* gene knock-out (^−/−^) phenotype (zebrafish deficient in its adaptive immune wing) to Spring Viremia Carp Virus (SVCV) infection. Remarkably, *Rag1^−/−^* zebrafish showed resistance to SVCV in an age-dependent manner, compared to their *Rag1^+/+^* counterparts. The analysis of the microarray data revealed that genes related to apoptotic functions, immune-related multigene families, and IFN-related components were constitutively upregulated in adult *Rag1^−/−^* zebrafish, thus preparing the host for the impending pathogen insult. In addition, in the absence of T and B cell functions, the *Rag1^−/−^* mice relied heavily on the infiltration of macrophages and natural killer cells, and IFNs for achieving a sustainable anti-viral state (García-Valtanen et al.; García-Valtanen et al.). Next, Kajla et al. report that heme peroxidase HPX15, which is found in the midgut of mosquito *Anopheles stephensi*, encourages development of *Plasmodium* parasite by suppressing local immune mechanisms. The orthologs of HPX15 are absent in non-*Anopheles* mosquitoes, insects, or human. Silencing of this gene *via* iRNA reduced the midgut parasite load in the mosquito, suggesting that interference with HPX15 gene can be exploited to contain the *Plasmodium* within the vector and minimize its dissemination.

There are five papers that take up a range of issues related to two key complement proteins, C1q and properdin. C1q is the first subcomponent of the complement classical pathway that binds to IgG or IgM containing immune complexes and a number of self and non-self target ligands *via* its globular (gC1q) domain. The gC1q domain is located C-terminal to a triple-helical collagen-like domain, which offers sites for interaction with C1r and C1s subcomponents, in addition to binding to its putative receptors, including calreticulin-CD91 complex (2). C1q is a versatile soluble charge pattern recognition molecule of the innate immunity. The gC1q domain is a heterotrimeric structure, which is composed of C-terminal ends of the three chains of C1q (A, B, and C chains). Individually expressed globular head (gh) modules of the three chains (ghA, ghB, and ghC) have been functionally characterized previously (3), which established the concept of structural and functional modularity within the heterotrimeric structure of the gC1q domain. However, in this volume, generation of a recombinant form of heterotrimeric gC1q domain is being reported by Gaboriaud et al. using a mammalian expression system (Moreau et al.), which is functionally active and its crystal structure mirrors that of the native gC1q domain generated *via* collagenase digestion of an intact human C1q molecule. Thus, this recombinant gC1q domain can be of great help in basic and clinical research on human C1q, in addition to being an excellent reagent/tool for the complement research community. On the continuing theme of gC1q domain, Pednekar et al. have examined the nature of interaction of the recombinant ghA, ghB, and ghC modules as well their substitution mutants with a gC1q putative receptor molecule, gC1qR. Another paper examines interaction between dendritic cell-specific intercellular adhesion molecule-3-grabbing non-integrin (DC-SIGN; CD209), C1q and gC1qR, and its implication on HIV-1 infection (Pednekar et al.). It sheds an interesting light on host–pathogen interaction involving three immune molecules and one pathogen component. C1q, by virtue of its ability to bind DC-SIGN, appears to suppress DC-SIGN mediated transfer of HIV-1 to CD4^+^ T cells while gC1qR appears to enhance trans-infection, raising the possibility that HIV-1 may recruit/exploit gC1qR in order to negate the protective effect of C1q (Pednekar et al.).

The final two papers in this issue report pattern recognition properties of human properdin, an upregulator of the complement alternative pathway. Properdin stabilizes C3bBb convertase in the alternative pathway, and its functions have largely been considered to be reliant on C3b deposition on the complement-activating surfaces (4). However, there are recent evidences to suggest that local production of properdin by immune cells including dendritic cells, neutrophils, and T cells may be geared at complement-independent functions of properdin. Kouser et al. report that human properdin can opsonize nanoparticles using its thrombospondin repeats 4 and 5 (TSR4 + 5) and mount a robust pro-inflammatory immune response *via* recruitment of NF-κB. One of the major findings reported in this paper is that a recombinant form of TSR4 + 5, expressed in tandem and coated on the nanoparticles, can act as an inhibitor of the alternative pathway, thus paving a way forward for *in vivo* preclinical trials of such nanoparticles for dampening exaggerated complement activation in a range of disease models (5; Kouser et al.). Al-Mozaini et al. have examined complement-independent interaction between human properdin and *Mycobacterium bovis* BCG. Contrary to their behavior when coated on nanoparticles, properdin, and recombinant TSR4 + 5 appear to exert an anti-opsonic effect on *M. bovis* BCG, thus, reducing micro-organism uptake by phagocytic cells (Al-Mozaini et al.). These two papers (Kouser et al.; Al-Mozaini et al.) appear to highlight that properdin can act as a soluble PRR, it does not always require the availability of C3b bound to target ligands, and that properdin on its own can modulate local inflammatory response. However, in the case of nanoparticles, properdin seems to enhance phagocytosis; whereas in the case of *M. bovis* BCG, it appears to reduce uptake: a contrasting and intriguing set of observations. Clearly, specific ligands need to be identified on *Mycobacterium* whose masking by properdin may interfere with surface receptors on macrophages that are used by this pathogen to enter its intracellular habitat.

## Author Contributions

UK reflected on the papers published within the volume and wrote the manuscript.

## Conflict of Interest Statement

The author declares that the research was conducted in the absence of any commercial or financial relationships that could be construed as a potential conflict of interest.
